# Hierarchical organization of long-range circuits in the olfactory cortices

**DOI:** 10.14814/phy2.12550

**Published:** 2015-09-28

**Authors:** Weiguo Yang, Qian-Quan Sun

**Affiliations:** 1 Department of Zoology and Physiology, University of WyomingLaramie, WY; 2 Graduate Neuroscience Program, University of WyomingLaramie, WY

**Keywords:** Interneuron, Piriform cortex, Neural circuit, Synapse

## Abstract

How sensory information is processed within olfactory cortices is unclear. Here, we examined long-range circuit wiring between different olfactory cortical regions of acute mouse brain slices using a channelrhodopsin-2 (ChR2)-based neuronal targeting approach. Our results provide detailed information regarding the synaptic properties of the reciprocal long-range monosynaptic glutamatergic projections (LRMGP) between and within anterior piriform cortex (aPC), posterior piriform cortex (pPC), and lateral entorhinal cortex (LEC), thereby creating a long-range inter- and intracortical circuit diagrams at the level of synapses and single cortical neurons. Our results reveal the following information regarding hierarchical intra- and intercortical organizations: (i) there is massive bottom-up (i.e., rostral–caudal) excitation within the LRMGP accompanied with strong feedforward (FF) inhibition; (ii) there are convergent FF connections onto LEC from both aPC and pPC; (iii) feedback (FB) intercortical connections are weak with a significant fraction of presumptive silent synapses; and (iv) intra and intercortical long-range connections lack layer specificity and their innervation of interneurons are stronger than neighboring pyramidal neurons. The elucidation of the distinct hierarchical organization of long-range olfactory cortical circuits paves the way for further understanding of higher order cortical processing within the olfactory system.

## Introduction

Olfactory cortex has been defined as those cortical regions receiving direct monosynaptic input from olfactory bulbs. Previous studies have reported the role of individual olfactory cortices in odor processing but the underlying cellular and synaptic mechanisms remain unclear (Wirth et al. 1998; Haberly 2001; Bernabeu et al. 2006; Mouly and Di 2006; Chapuis et al. 2013). Piriform cortex has been widely accepted as an associational rather than primary region of the olfactory cortex as discussed previously (Johnson et al. 2000; Haberly 2001; Neville and Haberly 2004; Zelano et al. 2005; Howard et al. 2009; Stettler and Axel 2009; Wilson and Rennaker 2010). Recently, imaging studies of human brain indicate aPC is more closely related to attention-related odor perception, while pPC tends toward related odor quality detection and odor categorization (Zelano et al. 2005; Howard et al. 2009). In vivo and in vitro studies of the rodent olfactory system have also begun to uncover the role of intracortical recurrent circuits within the PC (Zhan and Luo 2010; Franks et al. 2011; Luo 2011; Poo and Isaacson 2011). Another subdivision of olfactory cortex – LEC, immediately caudal to pPC in rodents – is suggested to serve as a higher order olfactory center via strong reciprocal connections with memory and emotion processing structures such as amygdala and hippocampus (Haberly and Price 1978; Agster and Burwell 2009; Xu and Wilson 2012; Chapuis et al. 2013). Multiple studies have defined the anatomical connections between aPC, pPC, and LEC (Luskin and Price 1983a,b; Burwell and Amaral 1998; Kerr et al. 2007; Agster and Burwell 2009), although quantitative information at synaptic levels remains elusive. Thus, it is still unclear how feedforward (FF) and feedback (FB) connections within these brain areas are organized and whether it is comparative to those in the neocortex.

To address this issue, we aimed to provide a detailed long-range circuit wiring diagram between and within different cortical regions involved in higher order olfactory processing. To build a detailed circuit diagram, one needs to measure functional synaptic connections between specific types of neurons. In the last few decades, electrophysiological recordings from connected cortical neurons in brain slices have demonstrated many intra- and interlaminar connections between specific neuronal subtypes (Deuchars et al. 1994; Markram 1997; Feldmeyer and Sakmann 2000; Thomson and Morris 2002; Thomson and Bannister 2003). However, one limitation is that it is difficult to study long-range connections between brain areas separated by distances on a scale of a millimeter or more because long-range projection axons are likely to be cut in a 300-*μ*m thick brain slice (Rocco and Brumberg 2007; Stepanyants et al. 2009). The ChR2-based circuit mapping approach overcomes this limitation (Petreanu et al. 2007; Wang et al. 2007). To the best of our knowledge, a study which provide systematic mapping of the entire olfactory cortex has not been done. This type of study is crucial for understanding how odors are decoded within the higher order olfactory cortices. Although a recent study by Hagiwara et al. (2012) investigated the associational circuits within the olfactory cortex, it mainly focused on the monosynaptic connections between anterior olfactory nucleus (AON) and PC, and intrinsic connections within PC. Similarly, a study by Luna and Morozov (2012) focused on input-specific excitation of specific neuronal targets within the pPC. Thus, our goal is to construct a map of synaptic connections within the PC, with information containing the probability and weight of monosynaptic connections in a bidirectional and layer-specific manner among major cell types of three olfactory cortices – aPC, pPC, and LEC.

## Methods

### Ethical approval

All procedures in this study conformed to the U.S. National Institutes of Health Guide to the Care and Use of Laboratory Animals and were approved by the IACUC and Institutional Biosafety Committee of the University of Wyoming.

### Animals

CD1 mice and GAD67-GFP (Δneo) mice (back-crossed on a CD1 background) were used in this study (Tamamaki et al. 2003; Jiao et al. 2006). All of mice were housed at the animal facility at the University of Wyoming in a temperature-controlled environment (72.2 ± 0.2°F) with a 12/12 h light–dark cycle (lights on at 6:00 am). Food and water were available ad libitum.

### Stereotaxic viral injection and normalization of virus expression

**Figure 1:**
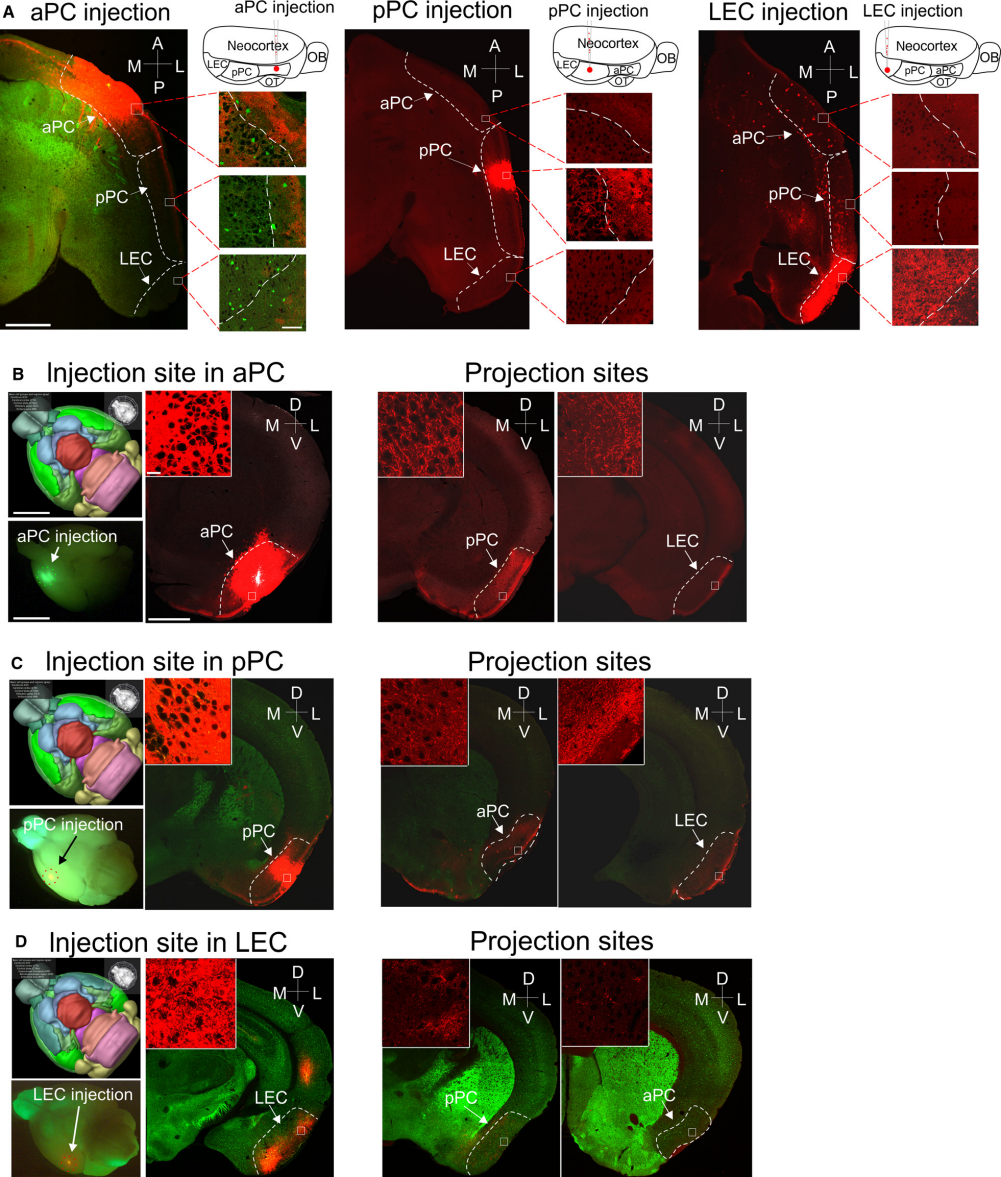
Verification of virus injection and projection in individual olfactory cortical areas. (A) Confocal images of 50-*μ*m thick horizontal slices showing virus-ChR2 injection sites in aPC, pPC, and LEC, respectively. Each injected cortical area was accompanied with a virus injection schematic (top right). In aPC injection, GAD67-GFP mouse was used, whereas for pPC and LEC injection, CD1 mice were used. The dash lines show the borderlines surrounding the individual cortical areas indicated by the arrows. The enlarged images on the right of each horizontal slice show the cytoarchitecture of each individual cortical area, with the dashed lines delineating the borderline between layer Ib and layer IIa. (B–D) Top-left panels: ventral views of mice brain from the Brain Explorer 2 (Allen Brain Institute) with PC or LEC highlighted in bright green; bottom-left panels: ventral views of mouse brains with AAV-ChR2 injection sites indicated by circled fluorescence in aPC, pPC, or LEC. The right panels were photomicrographs of 50 *μ*m coronal slices from the same brain showing injection (left) and projection (right) sites, respectively. The enlarged images in the inset boxes show fluorescent fibers (red) in either the injection or the projection areas. Notably, in LEC injections (D), both aPC and pPC showed weak fluorescence, indicating sparse back projections from LEC to the PC. However, many retrograde labeling red beads (red dots in the insets) were visible in both aPC and pPC, meaning that these labeled neurons sent axons to the LEC and this weak back projection was unlikely to be an artifact due to the severed fiber system. Scale bars (A) = 1 mm (low-magnification image) and 50 *μ*m (high-magnification panels); (B) = 3 mm (brain schematic and below image) and 1 mm (low-magnification image) and 10 *μ*m (high-magnification panel). The same scales were used for the remaining panels in the same manner.

Virus injection was performed in juvenile mice (male and female) at postnatal days 12–13 (P12–13). Mice were anesthetized with 2% isoflurane (vol/vol) and maintained with oxygenated 1% isoflurane throughout the surgery procedure. A small scalp incision (typically 1 × 1 cm^2^) was cut to expose the region of interest. The stereotactic coordinates for aPC, pPC, and LEC of P12–13 mice were AP +1700/LM 2700/DV 3600, AP -200/LM 3900/DV 4200, and AP -1600/LM 3800/DV 3900 (from Bregma, AP: anterior/posterior axis, LM: lateral/medial axis, DV: dorsal/ventral axis), respectively (Fig. 1A). These coordinates were derived from preliminary anatomical studies of 15 mice aimed at precisely targeting individual cortical areas (five mice for each region), which were shifted from the coordinates for adult mice (Paxinos and Franklin 2004) (see Fig. 1A–D). Mice exhibiting out-of-target virus injection or low-level viral expression were excluded from further experiments. AAV2/rh.10.CAGS.ChR2-venus and AAV2/1.CAG.hChR2 (H134R)-mCherry were purchased from the University of Pennsylvania Vector Core. One microliter of virus (either AAV-ChR2-venus or AAV-ChR2-mCherry, simplified as AAV-ChR2) was mixed with 1 *μ*L of red beads (for visualization) and injected into each mouse brain using a beveled glass micropipette (Drummond Scientific Co., Broomall, PA; Cat. # 5-000-2010) with a tip diameter 20–30 *μ*m fabricated using a P-97 pipette puller (Sutter Instrument Co., Novato, CA). Based on preliminary anatomical studies, we found a virus volume of 80–100 nL yielded stable (and strong local virus expression (spanning all layers) and long-range projection without invading subcortical structures for the majority of our slices (Fig. 1A–D). Those animals with inefficient or excessive invading nontargeted structures) viral expression were excluded from the current study. In three mice, an increase volume of virus (150–300 nL) was delivered into the LEC (one example of a 300 nL injection shown in Fig. 1A, right panel) to determine whether the weak back projection from LEC to PC was due to an insufficient amount of virus injection into the LEC. The virus injection protocol was normally completed within 2 min. The pipette was left in place for 5–6 min upon completion of an injection and was pulled out slowly. To allow sufficient viral expression, animals were given a 2- to 3-week recovery period following virus injection before performing any electrophysiological experiments (mice aged P28–P35). Injected mice were put back with the dam immediately after recovery from anesthesia in a separate cage. At P21, virus-injected pups were weaned and group housed in a separate cage until electrophysiological experiments were performed.

### Brain slices electrophysiology

Mice were deeply anesthetized with 2% isoflurane delivered in oxygen and decapitated. The brain was quickly removed and transferred into a chamber filled with ice-cold oxygenated aCSF for at least 10 min. The aCSF solution consisted of the following (in mmol/L): 126.0 NaCl, 2.5 KCl, 1.25 NaH_2_PO_4_, 1.0 MgCl_2_, 2.0 CaCl_2_, 26.0 NaHCO_3_, and 10.0 glucose. Horizontal slices (300-*μ*m thick) were cut as described elsewhere (Markopoulos et al. 2008) using a vibratome (TPI, St. Louis, MO) with dissecting solution containing (in mmol/L): 2.5 KCl, 1.25 NaH_2_PO_4_, 10.0 MgCl_2_, 0.5 CaCl_2_, 26.0 NaHCO_3_, 11.0 glucose, and 234.0 sucrose. Brain slices were incubated in a holding chamber containing 95% O_2_ and 5% CO_2_ bubbled aCSF at 35°C for 1 h before recording at room temperature (23°C). Borosilicate recording pipettes (3–6 MΩ) were filled with a Cs-based internal solution containing (in mmol/L): 120 cesium gluconate, 10 phosphocreatine-Tris, 3 MgCl_2_, 0.07 CaCl_2_, 4 EGTA, 10 HEPES, 4 Na_2_-ATP, and 1 Na-GTP (pH 7.35 adjusted with Cs-OH, 280 mOsm). Neurobiotin (0.5%, Vector Labs, Burlingame, CA; antibodies used from Santa Cruz Biotechnology, Dallas, TX: goat anti-rabbit HRP, sc-2030; Rb HRP, SC-2749; donkey anti-goat HRP, sc-2056) was added to the pipette solution for later morphological confirmation and relative laminar and rostrocaudal (RC) position of recorded cells.

**Figure 2:**
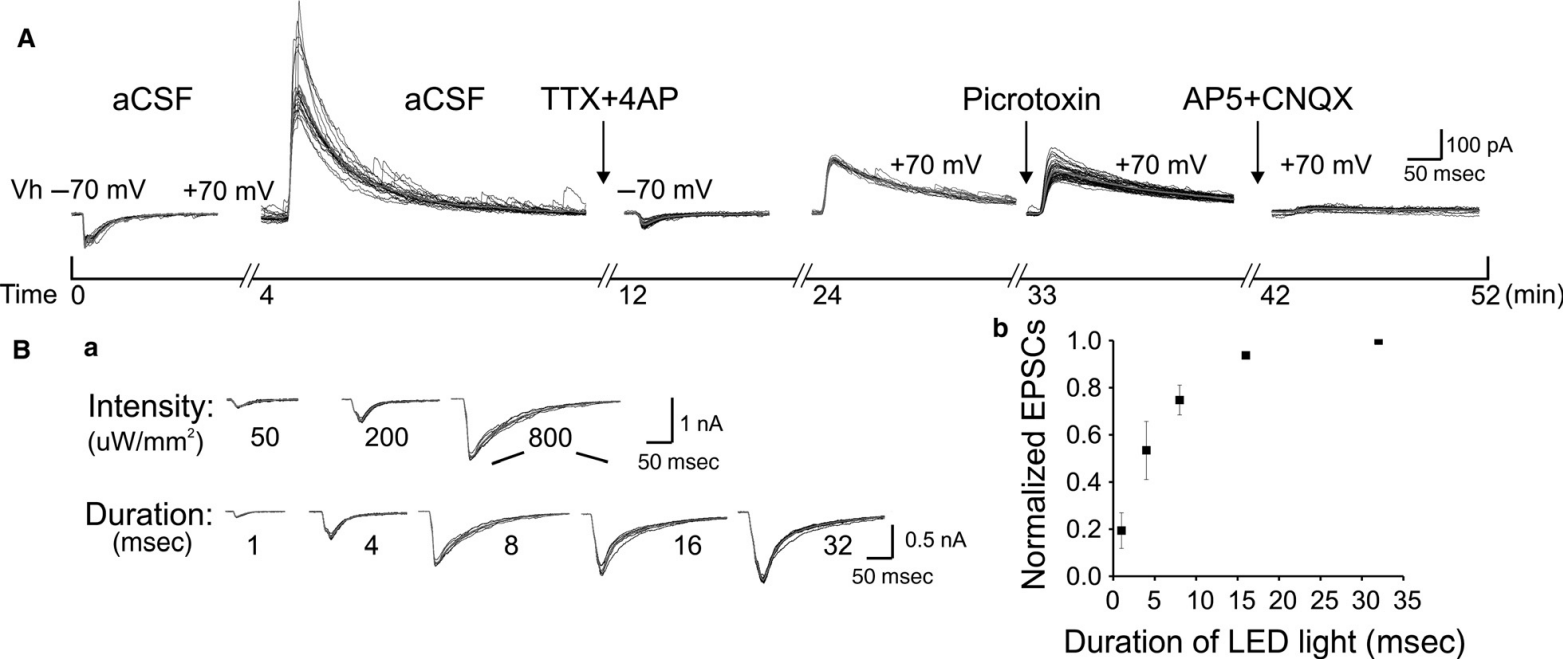
Pharmacological experiments and light stimulation parameters. (A) An example experiment showing optogenetic-induced AMPA and NMDA currents elicited in normal aCSF and in the presence of different glutamate and GABA agents in a timeline. Gray lines in the traces represent the mean. Note that the monosynaptic outward currents are eliminated by NDMA and AMPA antagonist (AP5 and CNQX, respectively). (B) *a*, Light-induced AMPA current traces with three increasing intensities (50, 200, and 800 *μ*W/mm^2^) and five increasing LED durations (1, 4, 8, 16, and 32 msec) at the same intensity (800 *μ*W/mm^2^); *b*, average plot of LED duration versus normalized EPSCs (normalized to the maximal AMPA currents). Responses derived from three neurons.

**Figure 3:**
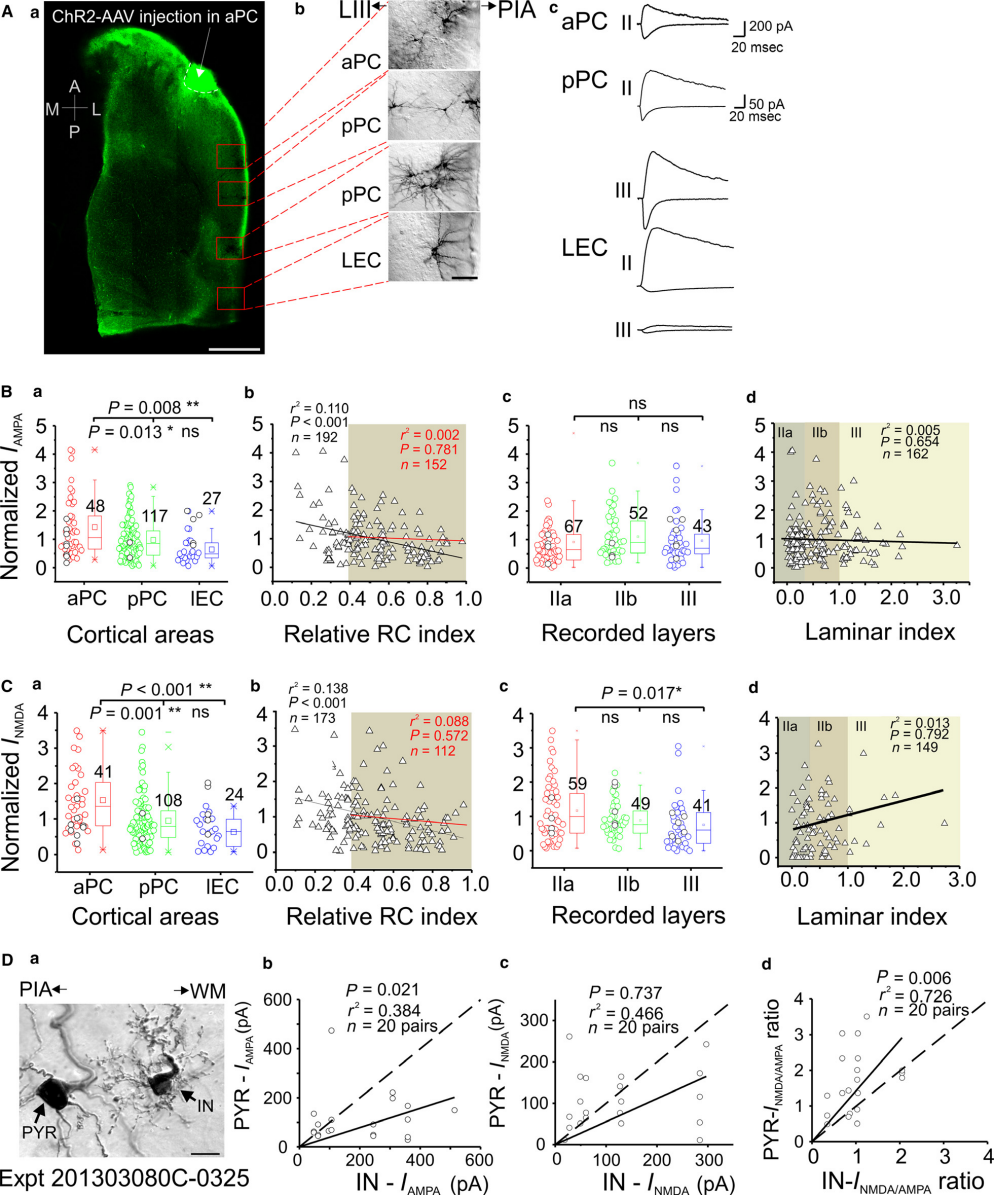
Whole-cell recordings of LRMGP-EPSCs in aPC AAV-ChR2-injected mice. (A) Representative experiment with AAV-ChR2 injection in the aPC; *a*, confocal image of 300-*μ*m thick horizontal slice showing the aPC injection site (white arrow) and the recording sites (red boxes); *b*, enlarged neurobiotin-labeled neurons; *c*, monosynaptic AMPA (downward) and AMPA + NMDA (upward) current traces recorded from neurons in three individual cortices. Note the differences of scale bars between aPC and pPC recordings. (B, C) Comparisons of monosynaptic AMPA and NMDA current strength (normalized to mean currents averaged from all recorded cells in the same slice, also apply to the following similar figures, see Methods section). The holding potential for AMPA and NMDA was –70 and +70 mV, respectively (also apply to the following figures). The shaded area in *b* shows linear fitting between two projection areas (red line, black line showing fitting with all recorded neurons, same for the following similar figures); shaded areas in *d* indicating different layers for this and other figures. (D) *a*, Bright-field image of an exemplar pair of neurobiotin-labeled IN and PYR recorded in pPC; *b*–*d*, three summary plots showing the comparisons of AMPA, NMDA, and their ratios, respectively, between INs and nearby PYRs recorded in pPC and LEC for aPC injection experiments. Scale bars (A) = 1 mm *(a)* and 50 *μ*m (*b*); (*D*) = 10 *μ*m. The same scales were used for their counterparts in the Figures 4, 5. *P < 0.05, **P < 0.01.

**Figure 4:**
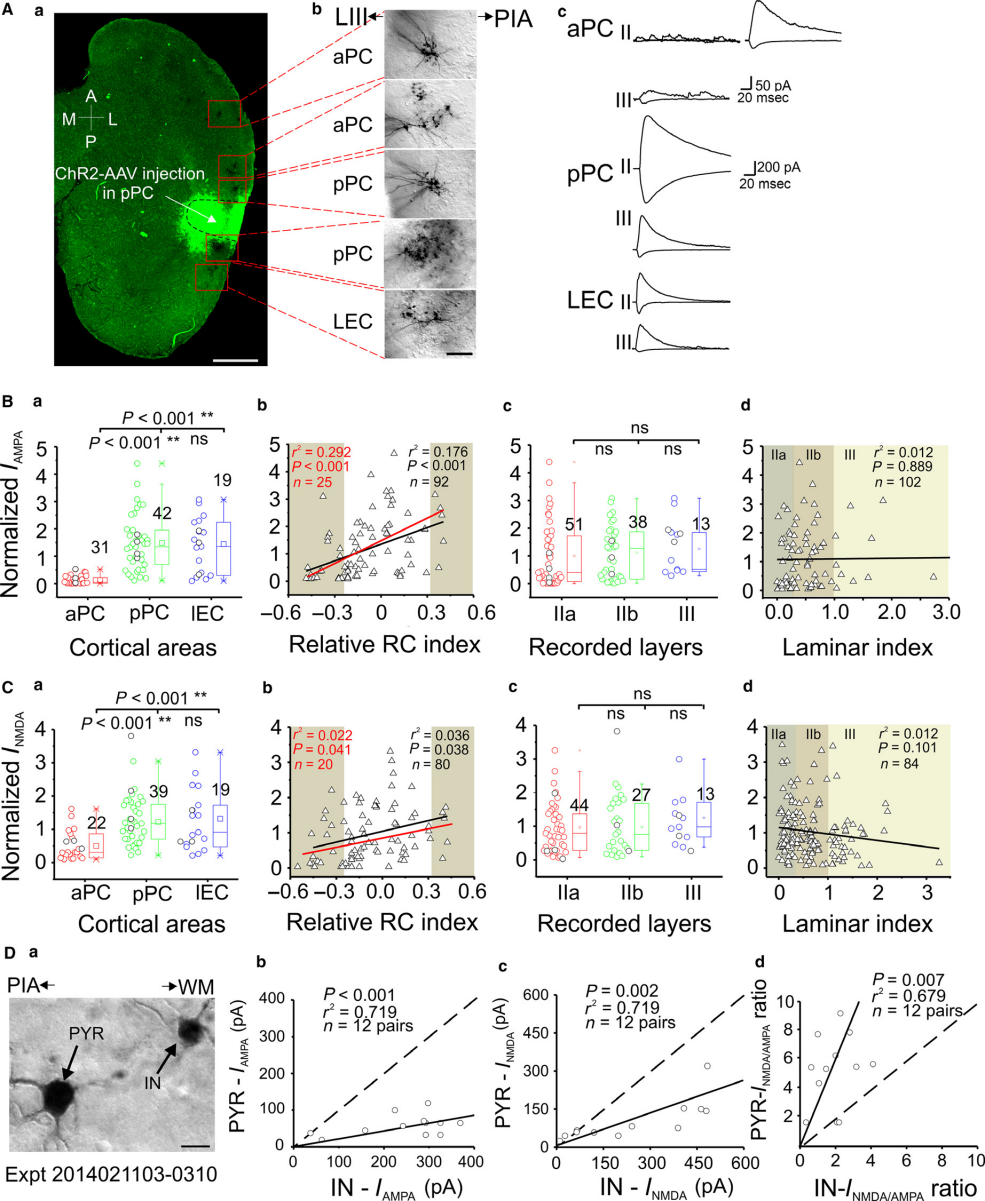
Whole-cell recordings of LRMGP-EPSCs in pPC AAV-ChR2-injected mice. (A) Representative experiment with AAV-ChR2 injection in the pPC; *a*, confocal image of 300-*μ*m thick horizontal slice showing the pPC injection site (white arrow) and the recording sites (red boxes); *b*, neurobiotin-labeled neurons; *c*, monosynaptic AMPA (downward) and AMPA + NMDA (upward) current traces recorded from neurons in three individual cortices. Note the differences of scale bars. (B, C) Comparisons of normalized monosynaptic AMPA and NMDA current strength. (D) *a*, Bright-field image of an exemplar pair of IN and PYR recorded in LEC with neurobiotin labeling; *b*-*d*, three summary plots showing the comparisons of AMPA, NMDA, and their ratios, respectively, between INs and nearby PYRs recorded in aPC and LEC for pPC injection experiments. *P < 0.05, **P < 0.01.

**Figure 5:**
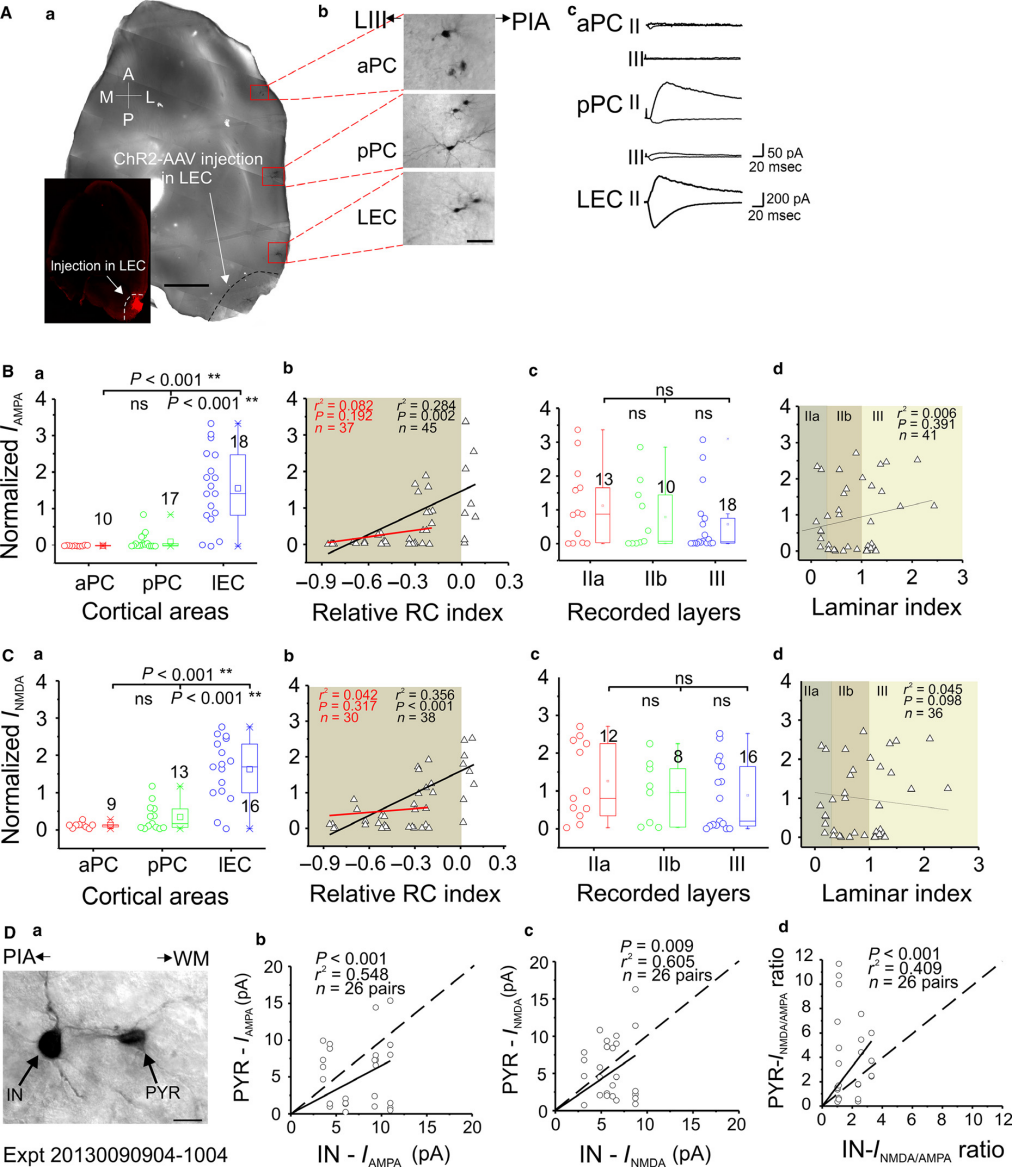
Whole-cell recordings of LRMGP-EPSCs in LEC AAV-ChR2-injected mice. (A) Representative experiment with AAV-ChR2 injection in the LEC; *a*, bright-field image of 300-*μ*m thick horizontal slice showing the LEC injection site (white arrow) and the recording sites (red boxes). The fluorescence of the recorded slice was quenched after DAB staining for neurobiotin, but an adjacent slice in the inset without staining shows that the injection site is located exactly in LEC; *b*, neurobiotin-labeled neurons; *c*, monosynaptic AMPA (downward) and AMPA + NMDA (upward) current traces recorded from neurons in three individual cortices. Note the differences of scale bars. (*B, C*) Comparisons of normalized monosynaptic AMPA and NMDA current strength. (*D*) *a*, Bright-field image of an exemplar pair of IN and PYR; *b*-*d*, three summary plots showing the comparisons of AMPA, NMDA, and their ratios, respectively, between INs and nearby PYRs recorded in aPC and pPC for LEC injection experiments. *P < 0.05, **P < 0.01.

Whole-cell recordings were obtained from either non-GABAergic, presumed glutamatergic pyramidal neurons (PYRs, including semilunar cells) or GABAergic interneurons (INs) in aPC, pPC, and LEC with the aid of infrared differential interference contrast imaging and GAD67-GFP fluorescent imaging. Recorded neurons were typically located 50–100 *μ*m below the surface of the recorded slice and had relatively intact dendritic arbors, verified by post hoc neurobiotin reconstruction (antibodies used from the Santa Cruz Biotechnology, Dallas, TX: goat anti-rabbit HRP, sc-2030; Rb HRP, sc-2749; donkey anti-goat HRP, sc2056). The holding potential was set at –70 mV to get the putative AMPA (*α*-amino-3-hydroxy-5-methyl-4-isoxazolepropionic acid)-mediated currents. After recording AMPA current traces, the holding potential was then set to positive 70 mV (to get a similar driving force as AMPA current) to get complex synaptic currents of AMPA and NMDA (*N*-methyl-d-aspartate) for another up to 20-trace recording. For offline analysis, peak of AMPA currents were subtracted from peak complex currents to obtain the putative NMDA-mediated current amplitude. The AMPA and NMDA peak currents were also used for calculation of their ratios (Wu et al. 1996). In a subset of experiments, picrotoxin was added to the bath to exclude the possibility of recording contamination from long-range inhibitory input (Figs.2A, 3–5B–C, *a*, *c*). The recorded cells were located at distal distances of at least 600 *μ*m from the epicenter of the virus injection, in an area devoid of any ChR2-positive cell bodies. In our “paired- recording” approach, the PYR and IN were located within 50 *μ*m from each other in the same layer. Recordings were made using a Multi-clamp 700B amplifier, filtered at 5 kHz, and sampled at 10 kHz. Data were accepted for analysis when serial resistance (Rs) was smaller than 30 MΩ and remained stable (<20% variation) throughout the recording. In this study, 32 mice were used for slice  recordings, including 15 aPC-injected mice, 10 pPC-injected mice, and 7 LEC-injected mice. On average, one horizontal slice per mouse was selected from 2 to 3 cut slices containing aPC, pPC, and LEC areas.

### Photostimulation and pharmacology

Monosynaptic glutamate release was triggered by illuminating ChR2 in the presynaptic terminals with a blue LED (488 nm, Thorlabs, LEDD1B) via a 63 ×  water immersion objective (pulled up from the focus by 0.5 mm) in the presence of 4-AP (100 *μ*mol/L, Sigma-Aldrich, St. Louis, MO) and tetrodotoxin (TTX, 1 *μ*mol/L, Alomone Labs, Jerusalem, Israel) (Petreanu et al. 2009; Cruikshank et al. 2010). With this method, all ChR2-positive synaptic terminals were presumably activated since the center of light is larger than the entire dendritic arbor of a typical pyramidal cell. In some experiments, picrotoxin (100 *μ*mol/L, TOCRIS) was added to examine if GABAergic inputs were involved in long-range projection (Fig. 2A). In a subset of experiments, CNQX (20 *μ*mol/L) and AP5 (50 *μ*mol/L) were added to confirm the involvement of AMPA and NMDA currents, respectively (Fig. 2A). Different stimulus conditions were tested with respect to light duration (1, 4, 8, 16, and 32 msec) and intensity (50, 200, and 800 *μ*W/mm^2^, Fig. 2B). We chose an LED duration of 8 msec (including trigger delay time) and an intensity of 800 *μ*W/mm^2^ (measured at the immediate back of the objective with a handheld laser power meter, Edmund Optics), which reached approximately 75% of the maximum response from postsynaptically recorded neurons across a relatively large range of virus infection levels (Fig. 2B, *b*). Activation of LED light was triggered with a custom computer program. The whole-cell-recording sequence was alternated from rostral to caudal in one experiment to caudal to rostral in the next experiment.

### Histology and confocal imaging

After recording, brain slices were transferred to 4% paraformaldehyde (PFA, wt/vol) in 0.01 mol/L phosphate-buffered saline (PBS) and fixed overnight at 4°C. Slices were then washed with 0.01 mol/L PBS twice (10 min each) before covered under glass coverslips with DAPI mounting media (Invitrogen, Grand Island, NY) and sealed with clear nail polish. Two to three weeks after virus injection, mice were anesthetized with 2% isoflurane (vol/vol) and transcardiac perfusions were performed with 30 mL normal saline followed by 30 mL 4% PFA. Brains were removed after perfusion and put in 4% PFA overnight, then subsequently put in 30% sucrose overnight for dehydration before sequential whole-brain 40-*μ*m coronal or horizontal sections with a cryostat (Microm HM 505 E). Slices were mounted with mounting media containing DAPI and sealed with clear nail polish for confocal imaging. Images were acquired with a laser confocal microscope (LSM 710) using magnification of 10 ×  or 63 ×  oil-immersion lens with 1.25 numerical apertures. Excitation wavelengths were 587 and 515 nm for mCherry and Venus, respectively. Images were processed and analyzed with ZEN software (ZEISS, Germany) and image J 1.48r (NIH).

### Statistics and data analysis

**Figure 6:**
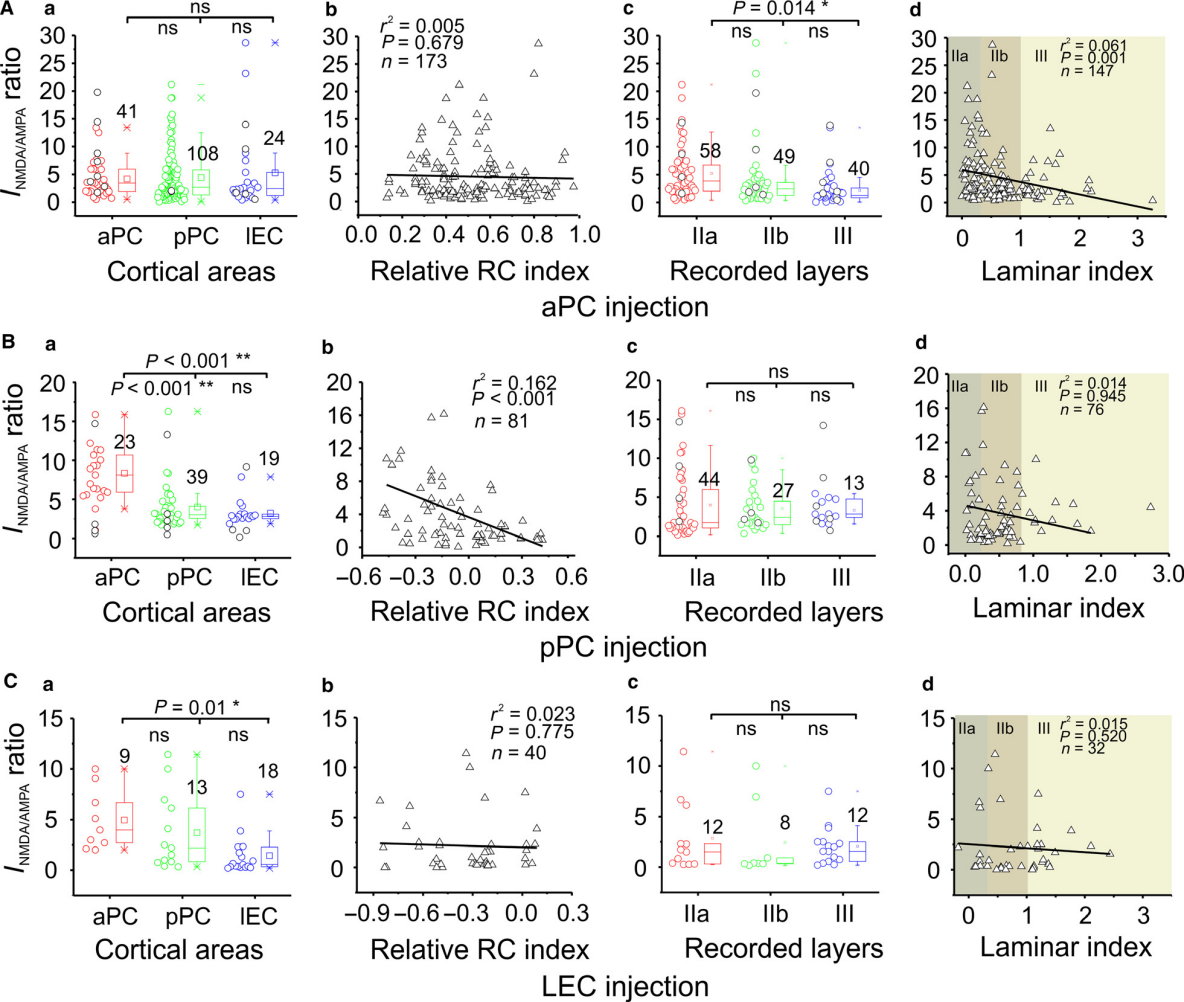
NMDA/AMPA ratios from monosynaptic current peaks of whole-cell recordings in different areas and layers. Data of NMDA/AMPA ratios from injection in aPC, pPC, or LEC were shown in *a*-*c*, respectively. All the data were from the recordings of glutamatergic neurons in three olfactory cortices in superficial layers. The total numbers of recorded neurons for each cortical area or layer are on the top of the individual graph. *P < 0.05, **P < 0.01.

Statistical analysis was performed using Origin. One-way analysis of variance (ANOVA) was used to examine statistical significance between groups. An unpaired two-sample *t*-test was used to compare current differences between two groups. A nonparametric Wilcoxon signed-rank test was used to compare current strength between INs and excitatory neurons. The box illustrations in the results indicated 25th and 75th percentiles (borders of a big box), mean (a small square within a box), median (a horizontal line within a box), 5th and 95th percentiles (short horizontal lines at both ends of a box), and 1st and 99th percentiles (at both extremes of a box). The standard for significant difference was defined as *P *<* *0.05. All graphical representations of data illustrations were presented as the mean ± SEM. An RC index was assigned to each neuron based on its location along the RC axis as revealed by post hoc neurobiotin labeling. The epicenter of virus injection was always defined as zero. For aPC injection, the border of LEC and medial entorhinal cortex was defined as 1 (Fig. 3B–C, *b*); for LEC injection, the border of AON and aPC as 1 (Fig. 5B–C, *b*). A soma position anterior to injection center was assigned a negative value. Each recorded neuron was also assigned a laminar index to analyze a layer-specific effect of projection. The border between layer Ib and IIa was arbitrarily defined as zero and the border between layer IIb and III as 1 (Figs. 6B–C, *d*). A soma position more superficial than zero was assigned a negative value. The amplitudes of light-evoked monosynaptic EPSCs were normalized by dividing each current peak by the mean value of EPSC peaks of all recorded cells in the same slice. An alternative normalization was to divide each current peak by the maximum current peak recorded in the same slice, which yielded the same result as above (see the raw data, provided as supplemental material, for detailed information about the normalization from recorded neurons).

## Results

### Functional connections from aPC to distal aPC, pPC and LEC

For long-range intracortical connections, we studied the projection (i.e., areas devoid of direct virus infections) but not the injection area of aPC, which we defined as “distal aPC” (see Methods section). Similar terminology (i.e., distal pPC or LEC) are used for other cortices, and “distal projection” region could be located either rostral or caudal to the injection site. We defined a feedforward (FF) pathway as projections from rostral to caudal (e.g., aPC **→ **pPC) and a feedback (FB) pathway as projections in the opposite direction (e.g., LEC **→ **pPC, LEC **→ **aPC,). We focused only on long-range monosynaptic glutamatergic projections (LRMGP), achieved by the application of TTX and 4-AP (plus picrotoxin in a subset of experiments) and careful examination of recorded slices to ensure recorded cells were located within a projection area (600 *μ*m or greater from the epicenter of the injection site and lacking ChR2-positive cell bodies).

First, we injected AAV-ChR2 in aPC to investigate the intracortical LRMGP within aPC as well as the FF-LRMGP from aPC **→ **pPC and from aPC **→ **LEC (Fig. 1A and B). An exemplar experiment is shown for the aPC injection (Fig. 3A). The confocal image (Fig. 3A, *a*) was taken after neurobiotin histological processing. The neurobiotin-labeled neurons (DAB stained) were shown on the right side to allow for identification of cell types and their positions (Fig. 3A, *b*). The intracortical LRMGP from rostral aPC to caudal aPC was much larger than those to pPC and LEC (Fig. 3A, *c*). For comparisons between different groups, we normalized the AMPA and NMDA currents by dividing the peak amplitude of each recorded cell by the mean amplitude of the same recorded slice to minimize the variability in the virus injection across different animals (Fig. 3B and C). Efforts were made to record a roughly equal number of neurons in each projection cortical region. Alternatively, we normalized the currents of each neuron to the strongest currents recorded in a single slice and the result was same as above (see supplemental materials). The results from recorded PYRs showed that intracortical aPC neurons received stronger intracortical LRMGP, while both pPC and LEC monosynaptic AMPA and NMDA synaptic strength were weaker compared to aPC **→ **aPC inputs (Fig. 3B–C, *a*). Surprisingly, there were no significant differences for both AMPA and NMDA currents between aPC **→ **pPC and aPC **→ **LEC synapses (Fig. 3B–C, *a*), indicating comparable associational monosynaptic fiber systems from aPC to both pPC and LEC. To quantitatively study if there was any attenuation of the LRMGP over long distance, we examined the correlations between the monosynaptic current peak amplitudes and the cells’ RC indexes (see Methods section). The long-range aPC projections studied here ranged from 0.6 to 6 mm (i.e., within an intracortical region and from rostral aPC to LEC). As to the neurons located in the projection regions (pPC and LEC), the results showed that there was no correlation between the normalized light-evoked AMPA and NMDA amplitudes and the locations (i.e., RC index) of the recorded neurons (Fig. 3B–C, *b*, shaded area, *r*^2^ = 0.002 and 0.088, respectively). These LRMGP also showed comparable strength (Fig. 3B–C, *b*, shaded area, *P *=* *0.781 and 0.572, respectively). The linear fitting of pooled data from all recorded PYRs indicated an inhomogeneous population (Fig. 3B–C, *b*, *P* < 0.001), which was apparently due to the dramatic weakening in the intercortical LRMGP strength of the aPC **→ **pPC and aPC **→ **LEC currents compared to the intracortical aPC **→ **distal aPC.

The PC is a three-layered paleocortex with clear anatomical segregation of sensory axons from main olfactory bulb (i.e., LOT) and intracortical projection axons (Haberly and Price 1978; Luskin and Price 1983a; Neville and Haberly 2004). It remains elusive whether intra- and intercortical monosynaptic connections have a functional layer and cell-type-specific preference. We recorded from all major types of PYRs in three layers/sublayers (layer IIa, IIb, and III) in aPC, pPC, and LEC. Laminar positions of recorded neurons were determined with a laminar index assigned for each recorded neuron (Figs. 6B–C, *d*). In the case of aPC AAV-ChR2 injection, we did not find significant differences between PYRs located in all three areas (layer IIa, IIb and III) with respect to AMPA and NMDA currents (Fig. 3B–C, *c–d*, respectively), with an exception of NMDA currents IIa versus III).

It has been reported recently that differential excitatory synaptic processing strategies have been employed by both principal neurons and INs in different layers in the PC (Suzuki and Bekkers 2011; Luna and Morozov 2012). We further explored whether FF and FB LRMGP in the three olfactory cortices studied differentially innervate PYRs versus INs. INs in layer I were not included in the dataset for PC recording since there were no PYRs in layer I (Neville and Haberly 2004); INs in layer I of LEC were included since principal neurons also exist in deep layer I (Canto and Witter 2012). INs were genetically labeled via GAD67-GFP expression and visualized by fluorescent microscopy. INs and PYRs located within 50 *μ*m were recorded as a “pair” for comparison of input strength from LRMGP (Figs. 5D). For aPC injection, the PYR and IN “pairs” were recorded from pPC and LEC (Fig. 3D, a showing one example from pPC). The results showed that INs received stronger monosynaptic AMPA strength than nearby PYRs but that the NMDA currents showed no bias to a particular type (Figs. 3D, *b–c*).

We also calculated the NMDA/AMPA ratios, which have been well established as a valid indicator of synaptic maturation (Wu et al. 1996). We found the NMDA/AMPA ratios were always skewed to PYRs (Figs. 5D, *d*), indicating that PYRs in these olfactory cortices might be more “silent” than INs, thus empowering them larger plasticity associated with the long-range excitatory innervation within these areas. In contrast, the relatively stronger AMPA currents in INs allow reliable FF and FB inhibition mediated by disynaptic glutamatergic recruitment of GABAergic neurons throughout major olfactory cortices.

### Functional connections from pPC to distal pPC, IEC, and aPC

We next injected AAV-ChR2 in pPC to investigate both the intracortical connections from pPC **→ **distal pPC, the FF pathway from pPC **→ **LEC and the FB pathway from pPC **→ **aPC (Fig. 1A and C). An exemplar experiment is shown for the pPC injection (Fig. 4A). The LRMGP from pPC to distal aPC were much weaker than those to distal pPC and LEC (Figs. 4A, *c* and B–C, *a*). In contrast to the aPC FF pathway above, the intercortical pPC → LEC projections showed similar monosynaptic AMPA (*P *=* *0.642) and NMDA strength (*P *=* *0.772) as the intracortical pPC → distal pPC connections with no significant attenuation in strength over long distance (Fig. 4B–C, *a–b*), suggesting a mechanism favoring FF information flow from associational cortex (pPC) to higher level olfactory center (LEC). This result suggested that long-range terminals located within LEC expressed sufficient ChR2 comparable to intracortical pPC after 2–3 weeks of virus expression, though several millimeters away from the injection region. It also eliminated the possibility that the gradient of AMPA and NMDA currents observed in aPC injection experiment was due to potential differential extent of AAV-ChR2 diffusion along the RC axis. Consistent with previous studies (Haberly 2001; Hagiwara et al. 2012), the FB projection strength of AMPA and NMDA from pPC → aPC was significantly weaker than the FF direction (Fig. 4B–C, *a–b*). Notably, two thirds of recorded neurons in aPC (61/92) showed no monosyanptic AMPA or NMDA currents from pPC synapses (excluded from the group analysis). These data suggest a unidirectional FF circuit organization from aPC → pPC and an inhomogeneous population of aPC neurons.

We further looked at the laminar projection pattern of pPC synapses. Similar to the results of the aPC injection experiments, we did not find any significant differences in AMPA and NMDA monosynaptic strength between layers IIa, IIb, and III in all three areas (Figs. 4B–C, *c–d*), indicating a general absence of layer-specific pPC synapses in the intracortical (pPC → distal pPC), FF (pPC → LEC), and FB (pPC → aPC) connections. The PYR and IN “pairs” in both aPC and LEC were also recorded (Fig. 4D). The group analysis showed the monosynaptic AMPA and NMDA inputs received by the INs were significantly higher than their nearby PYRs within same layers (Figs. 4D, *b–c*). Again, the NMDA/AMPA ratios remained higher in the PYRs compared to the nearby INs (Figs. 4D, *d*). These results were similar to aPC projections, which indicated reliable disynaptic inhibition mediated by LRMGP recruitment of GABAergic INs in both FF and FB pathways.

### Functional connections from LEC to distal LEC, aPC, and pPC

Finally, we injected AAV-ChR2 into LEC, which is known as a transitional six-layered cortical structure related to high-order olfactory information processing (Fig. 1A and D). An exemplar experiment is shown for the LEC injection (Fig. 5A). The inset shows an adjacent slice with AAV-ChR2-mCherry fluorescence indicating that the injection site located exactly within in center of LEC. Despite the strong intracortical responses in LEC → distal LEC, light-evoked AMPA and NMDA currents of the FB LEC synapses in pPC were significantly weaker. The LEC → aPC connection strength of AMPA and NMDA was similar to that of LEC → pPC projections (Fig. 5B–C, *a–b*). A large proportion of the recorded neurons in aPC (35/45) and pPC (26/43) did not show any APMA or NMDA response upon light stimulation (not included in the group analysis), which is in sharp contrast to the fact that a significant proportion of aPC excitatory neurons project to LEC (bead-labeled neurons, Fig. 1D, right panel, see also Fig. 3B–C, *a*). Together, these results suggest that the FB projections from pPC → aPC, and from LEC → aPC were weak, favoring a unidirectional FF fiber system. This, however, does not necessarily imply that the LEC is not important in modulating PC neuronal activities (see Discussion section).

Next, “pairs” of PYRs and INs were examined in aPC and pPC (Fig. 5D). The group analysis showed, similar to the pPC result, that INs received stronger monosynaptic AMPA and NMDA strength compared to the nearby PYRs (Fig. 5D, *b–c*). In addition, the NMDA/AMPA ratios were consistently higher in the PYRs in contrast to the INs (Fig. 5D, *d*). When comparing LRMGP innervation of INs between FF and FB pathways, the results showed that the FB connections were significantly weaker (*P *<* *0.001, *n *=* *46, unpaired two-sample *t*-test; for FB AMPA [pA, mean ± SE]: 35.4 ± 3.7, for FB NMDA, 79.2 ± 6.5; for FF AMPA, 292 ± 39.2, for FF NMDA, 284 ± 43.2). Our data thus suggest a stronger LRMGP to INs than nearby PYRs in both FF and FB olfactory cortical pathways.

### NMDA/AMPA ratios in different intracortical and intercortical connections

**Figure 7:**
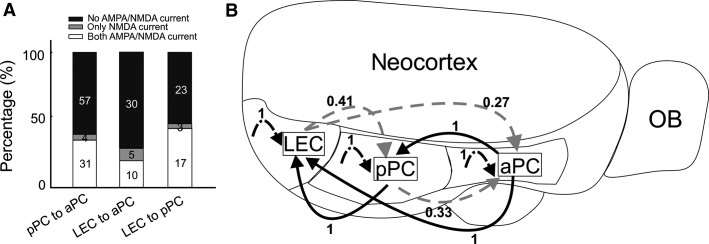
Connection probabilities. (A) Accumulative percentage of cells showing a particular response type (regarding NMDA/AMPA current) for each FB projection. Note that the “pure” silent synapses (gray bars) were found in a very small fraction of cells, while large fractions of recorded synapses show higher NMDA/AMPA ratios (white bars). (B) Diagram showing the connection probability of LRMGP determined by the ratios of the cell number with positive LRMGP value to the total recorded cell number within a cortical area. The black solid lines represented FF projections (RC direction) and gray dashed lines FB projections. Intracortical connection probabilities within each cortex were represented with black dot-dash lines. The number near the convex side of each line indicated the connection probability.

Next, we examined the NMDA/AMPA ratios for all of the recorded glutamatergic neurons in three individual cortices. For aPC AAV-ChR2 injection experiments, the NMDA/AMPA ratios showed no cortex-specific differences (Fig. 6A, *a–b*). The ratios between layer IIa and IIb also showed no differences; so did layer IIb and III (Fig. 6A, *c–d*). However, those between layer IIa and III did show a layer-specific difference (Fig. 6A, *c–d*). For pPC AAV-ChR2 injection experiments, the NMDA/AMPA ratios were significantly higher in the pPC or LEC → aPC FB pathways compared to intracortical pPC or the FF Ppc → LEC pathways (Fig. 6B, *a–b*), suggesting these FB synapses tend to be more “silent” than other synapses. Surprisingly, the FF and FB projections did not show any differences between layers (Fig. 6B, *c–d*), despite their different cytoarchitecture. For LEC AAV-ChR2 injection, the NMDA/AMPA ratios remained higher in aPC synapses, similar to the pPC injection case (Fig. 6C, *a–b*). No ratio differences were found between the three layers (Fig. 6C, *c–d*), suggesting an absence of layer-specific FB innervations from LEC → PC. Although different glutamatergic neuronal subtypes (e.g., semilunar cells) were located in a laminar-specific fashion (Suzuki and Bekkers 2011; Luna and Morozov 2012), our results suggest that these long-range intra- and intercortical connections within the PC showed little cell specificity. Notably, a significant number of synapses (57 of the 92 in pPC to aPC synapses, 30 of the 45 in LEC to aPC synapses, and 23 of the 43 in LEC to pPC synapses) did not show any light-induced AMPA or NMDA currents (Fig. 7A). More importantly, much higher NMDA/AMPA ratios were observed in the LEC and pPC → aPC synapses. Meanwhile, a small fraction of neurons showed “pure” silent synapses (i.e., only NMDA currents recorded, Fig. 7A). Together, these data suggest that abundant silent or near-silent synapses dominated the FB pathway. The LEC to pPC synapses also showed a trend toward higher NMDA/AMPA ratios, although not significantly different compared to other FB synapses (Fig. 6C, *a*). Overall, these results showed a different strategy in laminar processing in olfactory cortices as compared to other sensory modalities (e.g., somatosensory, visual, auditory cortex), as well as a significant fraction of presumptive silent synapses in FB long-range synapses.

### Connectivity probabilities within and between individual cortices

Inter- and intracortical connection probabilities were summarized in a circuit diagram (Fig. 7). The probability was defined as the ratio of the neuron number with AMPA-mediated monosynaptic current to the total neuron number recorded in an individual cortical area. All long-range (600–1200 *μ*m) intracortical connections within three cortical areas (aPC, pPC, and LEC) and the FF connection probabilities (Fig. 7, black solid lines) showed monosynaptic AMPA responses upon light stimulation with the probabilities equal to 1 (Fig. 7B, black dot-dash lines). However, the probability of the FB connection from LEC to pPC is only 0.41. Similarly, those of the FB connection from LEC and pPC to aPC are 0.27 and 0.33, respectively (Fig. 7B, gray dashed lines). In summary, using connection probability data we constructed an intracortical and intercortical LRMGP connectivity map based on AMPA and/or NMDA-mediated responses within and between aPC, pPC, and LEC.

## Discussion

Here, we provided a systemic LRMGP map within and between three primary olfactory cortical regions – aPC, pPC, and LEC (Fig. 7). Using optogenetic stimulation and whole-cell recording to examine the connection strength in single cortical neurons, we found FF connections (RC direction) are much stronger than FB connections, especially for LEC to PC projections which, to our knowledge, has not been reported before and thus provides a novel insight into how higher order olfactory center might modulate the lower hierarchical structure. In addition, these weaker and sparse FB connections may reflect the existence of silent synapses in the PC, supported by the higher NMDA/AMPA ratios of LEC and pPC synapses in aPC. This study also shows that the intracortical connections are much stronger than intercortical connections, with one exception of pPC synapses showing comparable connection strength in distal pPC and LEC. Notably, no apparent layer-specific projection pattern is found in the both FF and FB pathways in these three important olfactory cortical areas despite apparent cytoarchitectural differences across layers. Interestingly, INs always receive stronger glutamatergic connections than nearby PYRs for both FF and FB pathways, suggesting that cortical inhibition is likely to be activated no matter which pathway is activated.

AAV vector encoding ChR2 and fluorophore have been utilized in recent studies of monosynaptic circuit connections over long distances in neocortex (Mao et al. 2011; Yang et al. 2013). In our hands, monosynaptic responses can be reliably recorded in LEC neurons up to 6 mm distance in a RC FF direction (Fig. 3A, *a*). In addition, 27/27 recorded neurons in LEC received monosynaptic responses from long-range aPC neurons. Notably, the synaptic strength of connections is not significantly different in aPC → pPC compared to aPC → LEC (Fig. 3B, *a–b*). Together, these data suggest a theme of equally potent monosynaptic projections from aPC → pPC and LEC.

For pPC injection experiments, both FF (pPC → LEC) and FB (pPC → aPC) projections can be studied in single slices. The FF monosynaptic connections from pPC → LEC show comparable strength to the intracortical pPC circuit, while the FB projections show much weaker input strength, indicating that a unidirectional FF projection from pPC is favored. This is consistent with previous anatomical and functional studies (Haberly 2001; Hagiwara et al. 2012). One observation from pPC projection experiments is that the neurons in dorsal aPC but not ventral aPC receive projections from pPC. This has not been shown before but is supported by previous anatomical studies which show a stronger back projection from pPC to dorsal aPC compared to that to ventral aPC (Haberly 2001; Xu and Wilson 2012). These functional connection differences underlying pPC → dorsal aPC and pPC → ventral aPC suggest potential differential roles of the two subdivisions within aPC.

As to the LEC injection experiments, we found that the FB projection is much weaker compared to intracortical LEC synaptic connection strength. The result of much higher volume of virus injected in LEC (Fig. 1A, right panel) is similar to those with standard small volume injections in LEC (Fig. 5B–C, *a–b*), thus these data dismissed the possibility that this weak FB projection is simply due to inefficient virus injection in LEC neurons. In addition, the large fraction (23/43) of unresponsive aPC neurons might also suggest a heterogeneous population of recorded aPC neurons. Although a few LEC lesion experiments have suggested its role in top-down suppression of PC activity (Wirth et al. 1998; Bernabeu et al. 2006; Chapuis et al. 2013), the underlying circuit mechanisms remain unclear. Our results demonstrate that long-range olfactory cortical circuits are more likely constructed in a way facilitating unidirectional monosynaptic projections: from aPC → pPC, aPC → LEC, and pPC → LEC. On the contrary, the FB projections from pPC → aPC, LEC → pPC, and aPC were significantly weaker. In addition, unlike the thalamic relay in neocortex, projections within olfactory cortices seem organized equally across all layers and thus lack cell-specificity (discussed below), which is reminiscent of the equally distributed projection pattern from the olfactory bulb to PC. However, a caution on how to interpret these results should be raised, that is, the observations are made using bulk injection approach such that most cells located in different layers of the injected regions are transfected with ChR2. The possibility that there is still layer-specific projections cannot be entirely ruled out until results from approaches using layer-specific targeting strategy become available.

Substantial anatomical investigations have been performed to study layer (or sublaminae) specific associational projections in PC (Haberly and Price 1978; Luskin and Price 1983b; Burwell and Amaral 1998; Agster and Burwell 2009). Among these studies, an HRP-labeling study shows that the associative projections are mainly in layer Ib and III. This result is consistent with the patterns of virus projection in the current study (Fig. 1). However, it is unclear if cells located in different layers are differentially targeted. Interestingly, despite the anatomical segregation of intracortical projections, we found that almost all recorded cells located in different layers received similar LRMGP (with only one exception, Fig. 3C, *c*). This is in contrast to observations in neocortex, where pathway- and laminae-specific differences in fiber innervations have been consistently reported. In visual cortex, for example, FF and FB projections show a laminar and pathway-specific difference. Specifically, FF projections from lower- to higher hierarchy visual cortex terminate mainly in layer 4 while FB connections tend to avoid layer 4 (Rockland and Pandya 1979; Yang et al. 2013). In addition, thalamocortical projections and long-range projections from motor cortex to somatosensory cortex are all characterized by prominent laminar differences (Kuramoto et al. 2009; Mao et al. 2011; Hooks et al. 2013).

Previous anatomical studies in aPC and pPC have led to the hypothesis that RC FF projections accumulate in more caudally positioned pPC neurons (Haberly 2001), therefore these neurons integrate more associational information and might be well poised for perception of environmental, emotional, and memory-related odor information. However, functional synaptic data are quite limited. The hierarchy of LRMGP, as demonstrated in this study, suggests that there is a bottom-up (FF) excitation dominating information flow within the long-range excitatory circuits along the RC axis. Thus, these results are in overall agreement with the idea proposed by Haberly (2001). In addition, our results further the idea by showing that LEC cells receive even more associational FF information, due to convergent pPC- and aPC-originated FF projections. Notably, intracortical NMDA/AMPA ratios in aPC showed similar levels with those in the FF aPC → pPC and aPC → LEC (Fig. 6A, *a–b*), which were in sharp contrast to those in FB pathways and strengthened the abundance of silent synapses from pPC and LEC to aPC and from LEC to pPC. These presumptive silent synapses dominating FB pathways suggest that these silent synapses might be activated during odor discrimination and other nonemergent tasks so the perception of odor, first processed in olfactory bulbs, could be modulated by FB inputs from other associational cortices (e.g., pPC) and higher level olfactory centers (e.g., LEC) (Wirth et al. 1998; Bernabeu et al. 2006; Chapuis et al. 2013). Although silent synapses are prominent in an early developmental stage and usually diminish during adulthood, their existence in the olfactory cortex implies a more flexible plasticity for odor processing tasks, at least in juvenile mice used in this study. Interestingly, previous studies in rats showed silent synapses are abundant in the aPC for afferent inputs (from olfactory bulbs) at the early developmental stage (Franks and Isaacson 2005). Moreover, intracortical synapses in aPC remain plastic throughout adulthood (Poo and Isaacson 2007). Our study thus extends these findings to FB intercortical synapses from LEC. The existence of presumptive silent synapses in FB pathways revealed in this study might compensate the weakness of the FB long-range monosynaptic connection, thus endowing LEC a strong top-down modulation of PC activities. It is also worth mentioning that this study does not exclude the possibility of a top-down modulation of PC by other olfactory structures such as basolateral amygdala (Mouly and Di 2006).

Our results showed that the fibers in FF and FB pathways had stronger monosynaptic connections with INs than nearby PYRs, indicating dominance for inhibition in olfactory cortices two-way information control (Figs. 5D). In neocortex, FF inhibition shortens the integration window for acquiring synchronous input, and thus is suitable for stimulus feature extraction and detection (Gabernet et al. 2005; Cruikshank et al. 2007, 2010). Our results also showed that the LRMGP of INs in the FB pathway was significantly weaker compared to the FF pathway. Stronger inhibition in a FF pathway (RC) indicates a widespread synchrony coding theme throughout neocortex and paleocortex (Bruno 2011). In contrast, the weaker inhibition in FB pathway might modulate the temporal sensitivity of intra- and intercortical olfactory circuits and tune the firing probability by favoring asynchronous activities in these lower hierarchy cortical areas (Kremkow et al. 2010; Bruno 2011). This is also supported by LEC lesion experiments which resulted in facilitation of odor recognition and expression of c-fos (Wirth et al. 1998; Bernabeu et al. 2006).

Together, this study highlights the cortical circuit wiring differences at a monosynaptic level between the evolutionarily new neocortex and the primitive paleocortex. The construction of the excitatory synaptic map within and between olfactory cortices is a necessary step toward better understanding how higher order cortices decode olfaction.

## Supporting Information

**Data S1.** Excel sheets of a summary of original data.
